# Analysis of Cow-Calf Microbiome Transfer Routes and Microbiome Diversity in the Newborn Holstein Dairy Calf Hindgut

**DOI:** 10.3389/fnut.2021.736270

**Published:** 2021-10-25

**Authors:** Huan Zhu, Minna Yang, Juan J. Loor, Ahmed Elolimy, Lingyan Li, Chuang Xu, Weidong Wang, Shuxin Yin, Yongli Qu

**Affiliations:** ^1^Heilongjiang Provincial Key Laboratory of Prevention and Control of Bovine Diseases, College of Animal Science and Veterinary Medicine, Heilongjiang Bayi Agricultural University, Daqing, China; ^2^College of Science, Heilongjiang Bayi Agricultural University, Daqing, China; ^3^Mammalian NutriPhysioGenomics, Department of Animal Sciences and Division of Nutritional Sciences, University of Illinois, Urbana, IL, United States; ^4^Heilongjiang Provincial Key Laboratory of Environmental Microbiology and Recycling of Argo-Waste in Cold Region, College of Life Science and Technology, Heilongjiang Bayi Agricultural University, Daqing, China

**Keywords:** maternal transfer, hindgut microbiome, dairy calf, diversity, SourceTracker

## Abstract

Hindgut microorganisms in newborn calves play an important role in the development of immunity and metabolism, and optimization of performance. However, knowledge of the extent to which microbiome colonization of the calf intestine is dependent on maternal characteristics is limited. In this study, placenta, umbilical cord, amniotic fluid, colostrum, cow feces, and calf meconium samples were collected from 6 Holstein cow-calf pairs. Microbial composition was analyzed by 16S rRNA gene high-throughput sequencing, and maternal transfer characteristics assessed using SourceTracker based on Gibbs sampling to fit the joint distribution using the mean proportions of each sample with meconium as the “sink” and other sample types as different “sources.” Alpha and beta diversity analyses revealed sample type-specific microbiome features: microbial composition of the placenta, umbilical cord, amniotic fluid, colostrum, and calf feces were similar, but differed from cow feces (*p* < 0.05). Compared with profiles of meconium vs. placenta, meconium vs. umbilical cord, and meconium vs. colostrum, differences between the meconium and amniotic fluid were most obvious. SourceTracker analysis revealed that 23.8 ± 2.21% of the meconium OTUs matched those of umbilical cord samples, followed by the meconium-placenta pair (15.57 ± 2.2%), meconium-colostrum pair (14.4 ± 1.9%), and meconium-amniotic fluid pair (11.2 ± 1.7%). The matching ratio between meconium and cow feces was the smallest (10.5 ± 1%). Overall, our data indicated that the composition of the meconium microflora was similar compared with multiple maternal sites including umbilical cord, placenta, colostrum, and amniotic fluid. The umbilical cord microflora seemed to contribute the most to colonization of the fecal microflora of calves. Bacteria with digestive functions such as cellulose decomposition and rumen fermentation were mainly transmitted during the maternal transfer process.

## Introduction

The newborn hindgut microbiome plays important metabolic and nutritional functions (1), with one of its main roles being the development of the intestinal barrier (2) and the maturation of the innate immune system in early life (3). Available studies in humans, lambs, and foals have focused on understanding the hindgut microbiome when microbial colonization starts (4–6). However, few studies have focused on the calf microbiome (7–9).

Whether the fetal gut and the maternal uterus harbor a microbiome prior to delivery has long been controversial (10). For instance, a number of studies have reported that the uterus is sterile and that mammals are exposed to exogenous microorganisms for the first time only at birth (11, 12). However, in recent years, a microbiota has been detected in amniotic fluid, placenta, and umbilical cord of humans during pregnancy and hindgut of newborn calves prior to colostrum feeding (7, 13, 14). Consequently, these data have given rise to the hypothesis of vertical transmission of the microbiome from mothers to offspring (4, 6). Although the precise source of the newborn gut microbiome is not known with certainty (4), some scholars considered that the newborn meconium arises from intrauterine seeding (15–17). In bovine, Klein-Jöbstl et al. (18) and Alipour et al. (19) evaluated the possibility of colonization of the calf fecal microbiota, and concluded that it was maternally related.

SourceTracker script has been used to evaluate quantitatively the contribution of different maternal microbiome constituents in the meconium of the offspring (4). For instance, He et al. (4) working with humans explored maternal transfer characteristics by comparing the microbiome in meconium with that in various maternal sites and concluded that the microbiome in meconium was inoculated from amniotic fluid, feces, vaginal fluid, and saliva, with the amniotic fluid making the greatest contribution. It is unknown to what extent, if any, similar events occur in livestock species such as dairy calves.

We hypothesized that hindgut flora colonization of newborn dairy calves is strongly influenced by different maternal sources including placenta, umbilical cord, amniotic fluid, colostrum and feces. To address this objective, we screened 6 cow-calf pairs to analyze the contribution of the microbiome in the maternal feces, placenta, amniotic fluid, colostrum, and umbilical cord to the seeding of the meconium microbiome using 16S rRNA sequencing technology and SourceTracker software.

## Materials and Methods

Animal care and experimental procedures were approved by the Animal Welfare and Ethics Committee of Heilongjiang Bayi Agriculture University, DaQing, China. Animal care and handling followed the guidelines of the regulations of the Administration of Affairs Concerning Experimental Animals (State Science and Technology Commission of China, 1988).

### Experimental Animals

Fifteen 3- to 5-year-old pregnant Holstein cows were procured from a large-scale commercial dairy farm within a 2-week period in December 2020. Selection of cow-calf pairs followed published criteria (20): (1) single calf; (2) calving difficulty score <3; (3) dam's colostrum quality assessed by a bovine colostrometer (HT-113ATC, Hengan Electronic Technology Co., China) of >50 mg/mL of IgG; (4) dam produced at least 3.8 L of good-quality first colostrum; and (5) calf birth weight >36 kg. Related information about cow-calf is shown in Table 1. According to the selection criteria, a total of 6 Holstein cow-calf pairs (two heifers and four steers) were selected for this research. To reduce environmental, management, and seasonal bias, all cows were fed the same diets and calved in a group calving pen without assistance, and sampled by the same experienced veterinarian. Ingredient and nutrient composition of diets and forages are shown in Table 2.

**Table 1:** Screening information for cows and calves.

**Items**	**IgG in cows' colostrum (mg/mL)**	**Amount of colostrum produced by cows (L)**	**Calf birth weight (kg)**
Value	51.15 ± 0.64	4.05 ± 0.18	41.67 ± 1.54

**Table 2:** Ingredients and chemical composition of dietary treatments of post-perinatal period dairy cow.

**Items**	**Value**
**Ingredient**	**%**
Corn silage	62.24
Oat grass	18.67
Wheat gluten	0.91
Soybean meal	3.63
Rice bran meal	0.78
Cottonseed meal 46%	3.95
Corn germ meal (sol.)	5.40
DDGS (distiller dried grains with solubles)	3.91
Powder	0.50
Total	100
**Nutrient levels**	
Dry matter in dairy ration (DM, %)	51.42
NE${}_{\text{L}}^{3}$, Mcal/kg	1.41
Crude protein (CP, %)	15.30
Crude fat (EE, %)	2.70
Starch, %	19.20
Neutral detergent fiber (NDF, %)	46.90
Acid detergent fiber (ADF, %)	33.40
Ash, %	6.96
Ca, %	0.46
P, %	0.30

### Sample Collection

During the second stage of labor when amniotic fluid vesicles were clearly visible and intact, a 60 mL sterile syringe was used to puncture these vesicles wearing sterile surgical gloves to harvest 50 mL of amniotic fluid that were subsequently deposited in two sterile tubes (6). Samples of placenta, umbilical cord, colostrum, cow feces, and meconium were collected aseptically within 1 h after delivery. After the natural delivery of the placenta, veterinarians wearing masks and sterile gloves collected two 1 cm^3^ slices from different regions of the placenta and umbilical cord using sterile scalpels (15), rinsed with physiologic saline. Once the placenta dropped on the ground, sampling stopped and the cow-calf were removed from the experimental animal. When sampling the umbilical cord, care was taken to avoid collecting at the site where the cord blood vessels pass through to prevent contamination of the sample by blood. During the colostrum collection process, the teats of the cows and surrounding areas were cleaned with sterile water, and then scrubbed with 75% ethanol by veterinarians wearing masks and sterile gloves. The first few drops of colostrum (~5 mL) were discarded, and the colostrum samples (50 mL) were collected into two sterile tubes (21). Considering the non-invasive nature of the sampling, cow feces and calf meconium were all collected from the rectum of the cow and calf, respectively, by veterinarians wearing sterile gloves. Meconium samples were collected before colostrum was fed to calves just after birth. Approximately 20 g were placed into each of two sterile tubes (19). All samples were stored temporarily in liquid nitrogen after collection and transported promptly to a −80°C freezer until analysis.

### DNA Extraction

Frozen samples were thawed at room temperature and total DNA extracted from each 1.5 mL sample of colostrum and 0.5 g sample of placenta, umbilical cord, amniotic fluid, cow feces and meconium using a CTAB (modified cetyltrimethylammonium bromide) method (22). The purity and concentration of DNA were assessed by agarose gel electrophoresis. A suitable amount of sample DNA was taken in a centrifuge tube and diluted with sterile water to 1 ng/μL. After extraction, the integrity of the DNA was detected by 1% agarose gel electrophoresis, and the concentration and purity of DNA detected by a NanoDrop 2000 (Thermo Fisher Scientific, United States). The isolated DNA was kept at −20°C until processing.

### 16S rRNA Amplification and Sequencing

Diluted genomic DNA was used as the template, and the bacterial V4 hypervariable region of 16S rDNA was amplified by PCR using specific primers with barcodes, Phusion^®^ High-Fidelity PCR Master Mix with GC Buffer from New England Biolabs, and a high-efficiency high-fidelity enzyme according to the selection of the sequencing region. The primer pair was 515F (5′-GTGCCAGCMGCCGCGGTAA-3′) and 806R (5′-GGACTACHVGGGTWTCTAAT-3′). The PCR products were multiplexed in a single pool in equimolar amounts and then detected by electrophoresis using 2% agarose gel electrophoresis after full mixing. The target bands were recovered using a gel recovery kit provided by Qiagen, and a TruSeq^®^ DNA PCR-Free Sample Preparation Kit was used for amplicon library preparation. All PCR reactions were carried out in 30 μL reactions, 0.2 μM of forward and reverse primers, and about 10 ng template DNA. Thermal cycling consisted of initial denaturation at 98°C for 1 min, followed by 30 cycles of denaturation at 98°C for 10 s, annealing at 50°C for 30 s, and elongation at 72°C for 30 s. Finally 72°C for 5 min. After quantification of the library with a Qubit 2.0 Fluorometer (Thermo Fisher Scientific Inc.) and quantitative PCR, sequencing was conducted using a NovaSeq6000 platform (Illumina, San Diego, CA, United States).

### Sequence Analyses

According to the barcode sequence and PCR amplification primer sequence, each set of sample data was separated from the accessory data. After the barcode and primer sequences were trimmed, FLASH software (V1.2.7) (23) was used to assemble reads that were barcode and primer free to obtain the raw tags (24). QIIME software (V1.9.1) (25) was used to filter out low-quality tags, detect sequences by comparison with the species annotation database, and remove chimeras. Lastly, the effective tags were retained for further analysis (26). Uparse software (V7.0.1001) was used to cluster all tags effectively to operational taxonomic units (OTUs) based on 97% identity of the sequences (27). The Mothur method and SSUrRNA database of SILVA132 (28, 29) were used to select and annotate the representative OTUs with the highest frequencies of occurrence for taxonomic information. Alpha and beta diversity were analyzed with QIIME software (V1.9.1). All graphs were drawn with R software (V4.0.3). SourceTracker software was used to predict the likely origin of the meconium microbiome using the maternal microbiome communities as potential “sources” and the meconium microbiome communities as “sink.” LEfSe software was used to perform LDA effect size (LEfSe) analysis, and the default LDA Score filter value was 4.

### Statistical Analyses

Data regarding composition of different samples were all analyzed for statistical significance *via* R software (V4.0.3). Differences between two groups were analyzed using Wilcoxon tests, and Tukey's test and the Wilcoxon test were selected if there were differences among more than two groups, and the confidence level was 0.05. Principal coordinate analysis and Permutational multivariate analysis of variance (PERMANOVA) were performed based on the weighted and unweighted UniFrac distances to evaluate the structural difference in the microbiota between different sample groups.

## Results

### Alpha Diversity of the Microbiome Community in Meconium and Maternal Samples

**Figure 1:**
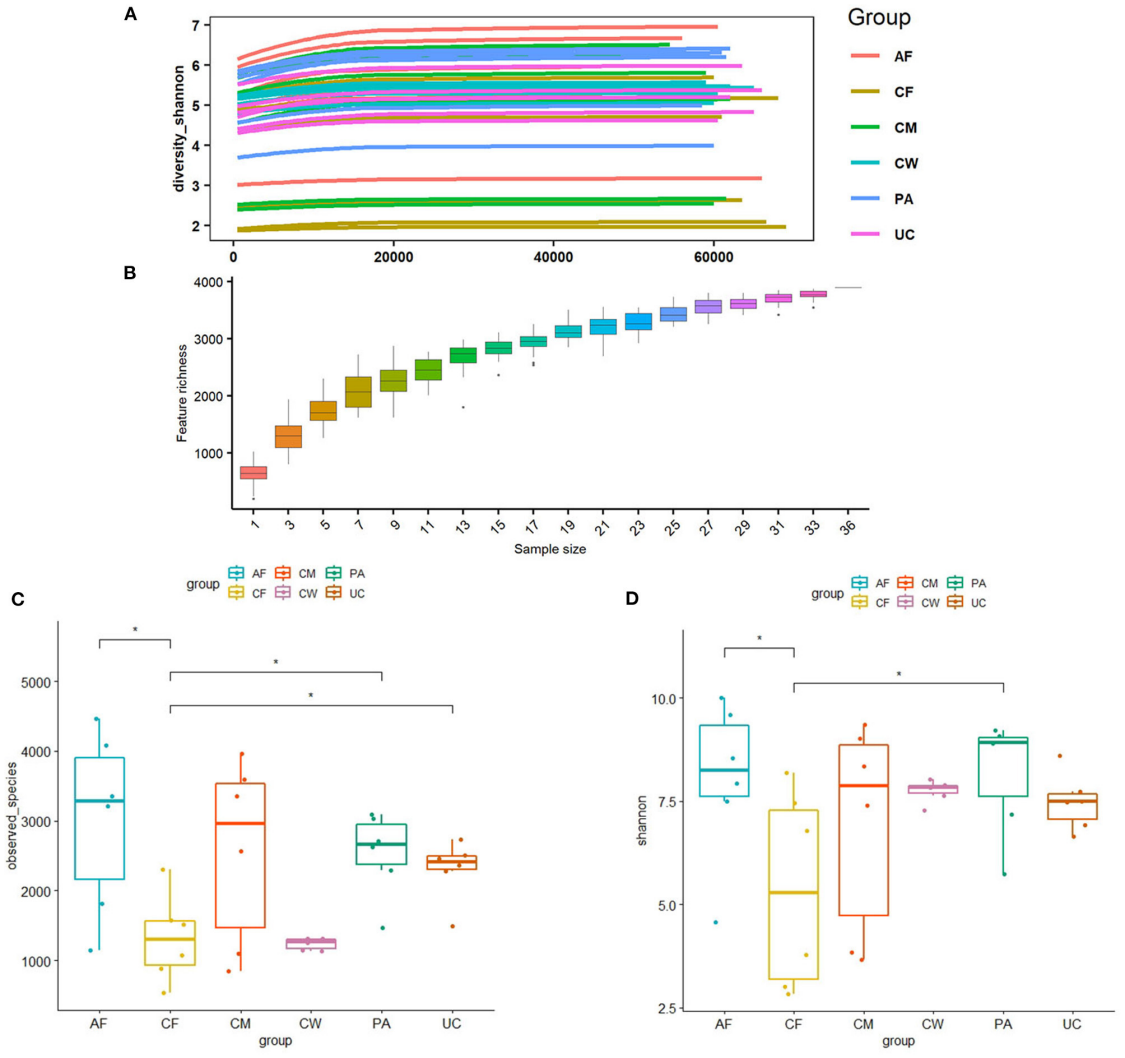
**(A)** Shannon diversity sparse curves, **(B)** box plot dilution curves, **(C)** box plot of the Shannon diversity index, and **(D)** observed characteristic index of all samples. AF, cow amniotic fluid; CF, calf meconium; CM, cow colostrum; CW, cow feces; PA, cow placenta; UC, cow umbilical cord.

Thirty-six examined samples were used as input for NovaSeq6000 to generate 2,260,449 high-quality sequencing reads at the genus level. Shannon, inverse Simpson, and Chao 1 estimator values for genera are also shown in Supplementary Table 1. The Shannon diversity curves leveled off, suggesting that the sequencing depth was enough to capture representative microbial diversity (Figure 1). The Shannon diversity index values varied in different groups (colostrum, 6.93 ± 2.34; meconium, 5.34 ± 2.19; cow feces, 7.76 ± 0.24; umbilical cord, 7.49 ± 0.62; placenta, 8.17 ± 1.29; amniotic fluid, 8.03 ± 1.77). Pairwise comparison by Wilcoxon test on Shannon diversity indexes for meconium and maternal parts (*P* < 0.05; Table 3). The coverage depth ranking is shown by rank–abundance curves; the OTU curve represented higher microbial diversity and richness (4).

**Table 3:** Wilcox test of alpha diversity index for meconium and maternal parts.

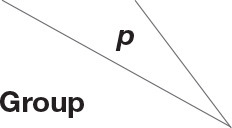 **Index**	**Observed_species**	**Shannon**	**Simpson**	**Chao1**	**ACE**
CF - AF	0.026^*^	0.041^*^	0.15	0.026^*^	0.026^*^
CF - CM	0.13	0.18	0.2	0.13	0.13
CF - CW	0.753	0.093	0.014^*^	0.82	0.82
CF - PA	0.026^*^	0.041^*^	0.065	0.015^*^	0.0087^**^
CF - UC	0.026^*^	0.13	0.31	0.026^*^	0.015^*^

*PA, placenta; UC, umbilical cord; AF, amniotic fluid; CM, colostrum; CW, cow feces; CF, calf meconium*.

* *p < 0.05*,

*p < 0.01.

### Beta Diversity of the Microbiome Community in Calf Meconium and Maternal Samples

**Figure 2:**
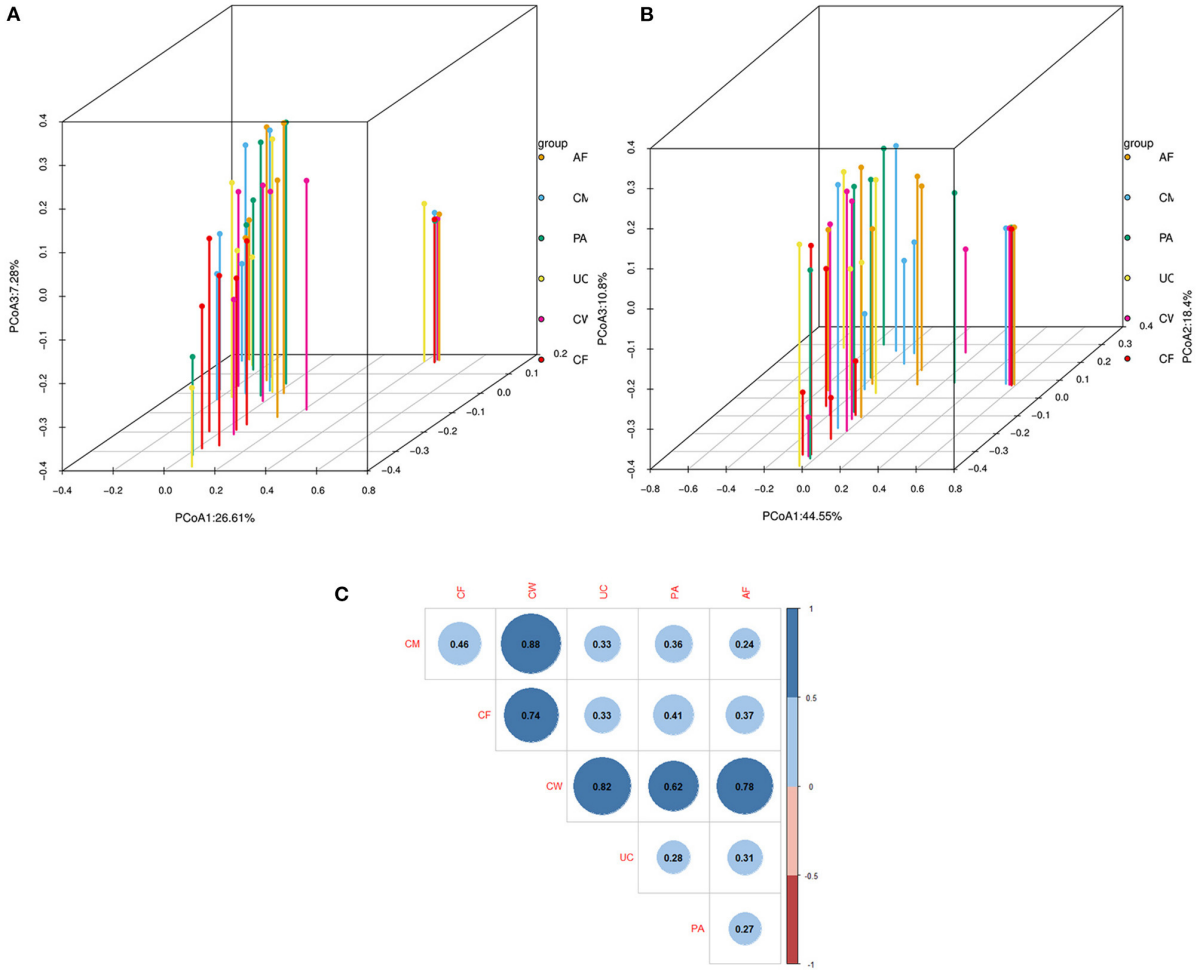
Dissimilarity-based multivariate analyses of microbiome communities of different sample types. Score 3D plots of principal coordinates analysis (PCoA) of different microbiome communities based on **(A)** weighted and **(B)** unweighted UniFrac distances. **(C)** Bray-Curtis dissimilarity matrix calculated based on microbial abundance patterns of operational taxonomic units (OTUs) of different sample types (Blue: positive, red: negative. The larger the absolute value is, the larger the area of the circle). The smaller the value of different degree indexes is, the higher the similarity (i.e., the difference between samples). AF, cow amniotic fluid; CF, calf meconium; CM, cow colostrum; CW, cow feces; PA, cow placenta; UC, cow umbilical cord.

Differences in the microbiome structure among the meconium and different maternal samples were evaluated by PCoA [permutational multivariate analysis of variance (PERMANOVA) by Adonis (Table 4)] and Bray-Curtis dissimilarity (Figure 2). The maternal feces clustered distinctly on the weighted and unweighted UniFrac. The weighted and unweighted UniFrac distance score 3D plot both showed that the meconium samples obviously clustered together (Figures 2A,B). In the weighted bray_curtis_dm matrix, the intergroup distance between meconium-placenta, meconium-umbilical cord, meconium-amniotic fluid, and meconium-colostrum were lower (0.33–0.46), and those between meconium-cow feces were higher (0.74). The differences between cow feces and other groups of samples were also higher (0.62–0.88, Figure 2C).

**Table 4:** PERMANOVA assessment of differences in bacterial community structure between two groups by Adonis.

**Group**	**Mean squares**	**Variation (R2)**	**P_adj_BH**
AF-UC	0.4878	0.1821	0.0255^*^
AF-CM	0.1707	0.0549	0.8530
AF-CF	0.7691	0.2175	0.0183^*^
AF-CW	1.7691	0.5539	0.006^**^
AF-PA	0.4215	0.1557	0.0808
UC-CM	0.6941	0.2206	0.006^**^
UC-CF	0.5847	0.2041	0.0491^*^
UC-CW	1.7668	0.6531	0.006^**^
UC-PA	0.2956	0.1412	0.1211
CM-CF	0.8698	0.2232	0.0131^*^
CM-CW	1.9119	0.5313	0.0129^*^
CM-PA	0.6088	0.1930	0.0563
CF-CW	1.9045	0.5573	0.006^**^
CF-PA	0.8579	0.2655	0.01^**^
CW-PA	1.0281	0.4991	0.006^**^

*PA, placenta; UC, umbilical cord; AF, amniotic fluid; CM, colostrum; CW, cow feces; CF, calf meconium*.

* p < 0.05, ^*^p < 0.01.

### Composition and Difference Analysis of the Microbiome Community in Meconium and Maternal Samples

**Figure 3:**
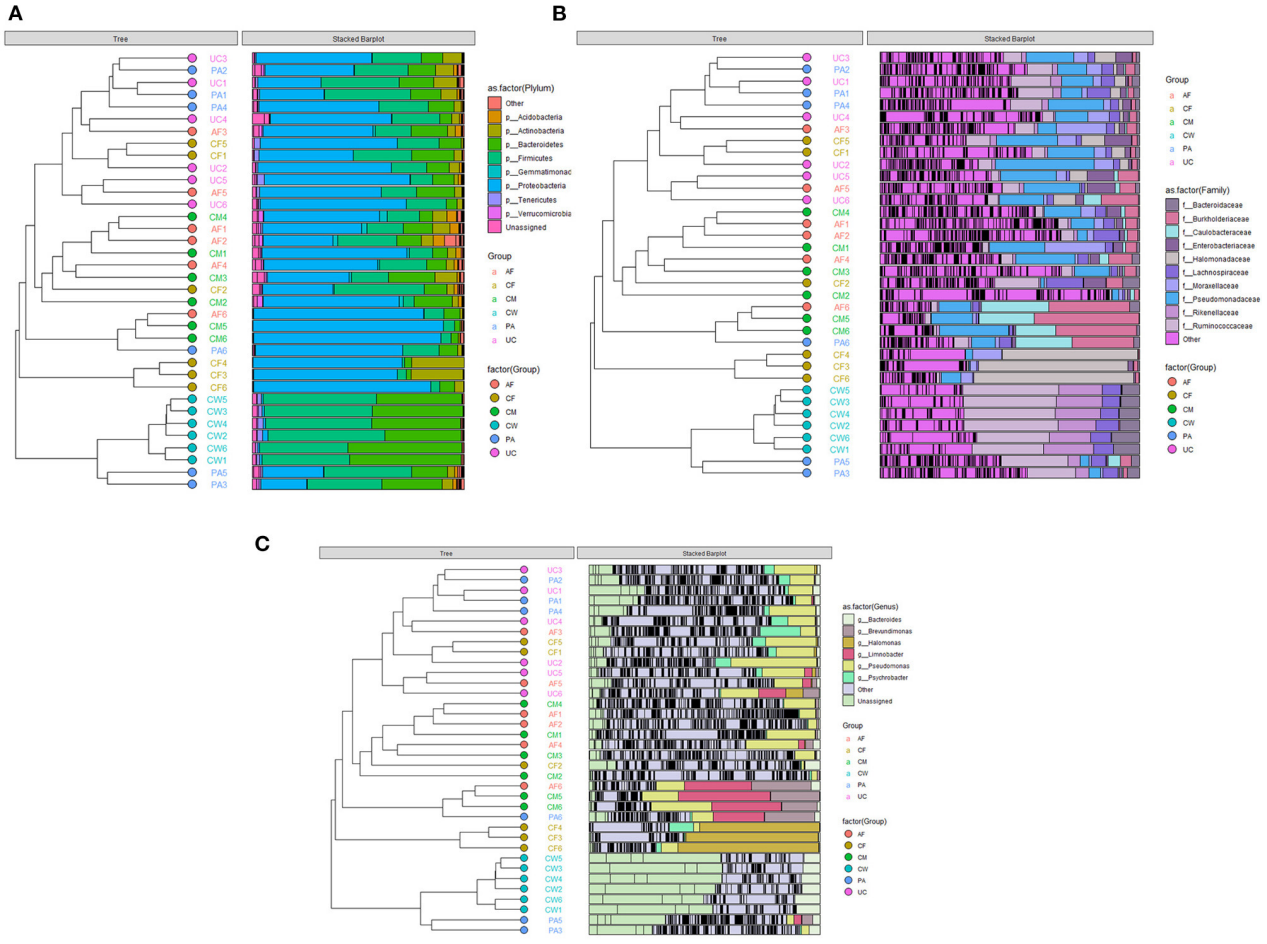
Clustering graph based on Bray-Curtis distance and stacked bar charts showing the microbiome compositions of the six types of samples at the **(A)** phylum level, **(B)** family level, and **(C)** genus level. AF, amniotic fluid; CF, calf meconium; CM, colostrum; CW, cow feces; PA, placenta; UC, umbilical cord.

The relative abundance and clustering characteristics of bacteria at the phylum, family, and genus levels from different sample types are shown in Figure 3. At the phylum level, the relative abundance of *Proteobacteria* in six parts, *Firmicutes* in five parts except colostrum, *Bacteroidetes* in amniotic fluid, placenta and cow feces were all >10%, and *Firmicutes* in colostrum and *Bacteroidetes* in colostrum, umbilical cord and calf feces were all close to 10, 9.8, 9.86, 9.76 and 8.94%, respectively (Figure 3A). The top represented bacterial families identified in the placenta, umbilical cord, and amniotic fluid were *Pseudomonas, Moraxella*, and *Ruminococcus* (Figure 3B). The colostrum contained mainly *Burkholderiaceae, Caulobacteraceae*, and *Pseudomonadaceae* families, and the meconium was dominated by *Halomonadaceae, Moraxellaceae*, and *Pseudomonadaceae* families. Lastly, cow feces contained mainly *Ruminococcaceae, Rikenellaceae, Lachnospiraceae*, and *Bacteroidetes* families. The microbial composition was dominated by *Bacteroidetes, Brevundimonas, Halomonas, Limnobacter, Pseudomonas*, and *Psychrobacter*.

**Figure 4:**
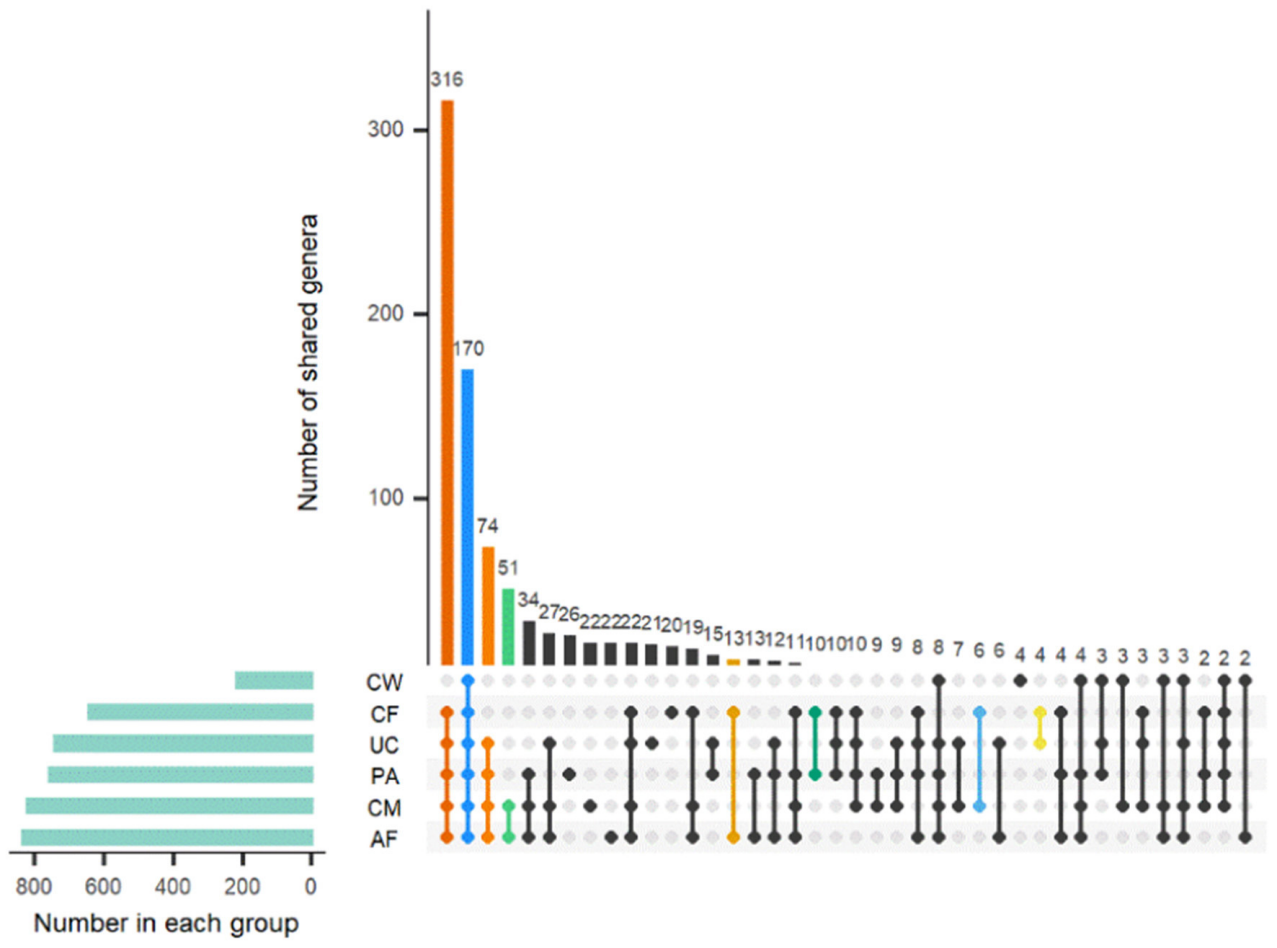
Distribution of OTUs shared among different sample types. AF, amniotic fluid; CF, calf meconium; CM, colostrum; CW, cow feces; PA, placenta; UC, umbilical cord.

Sample type-specific OTUs (detected exclusively in one sample type) were identified in all sample groups at the genus level (Figure 4). There were 26, 22, 22, 21, 20, and 4 type-specific OTUs in the placenta, colostrum, amniotic fluid, umbilical cord, meconium, and cow feces, respectively, and 170 OTUs shared by all parts were found (Figure 4). OTUs found exclusively in one type of sample at genus level are shown in Supplementary Table 3.

**Figure 5:**
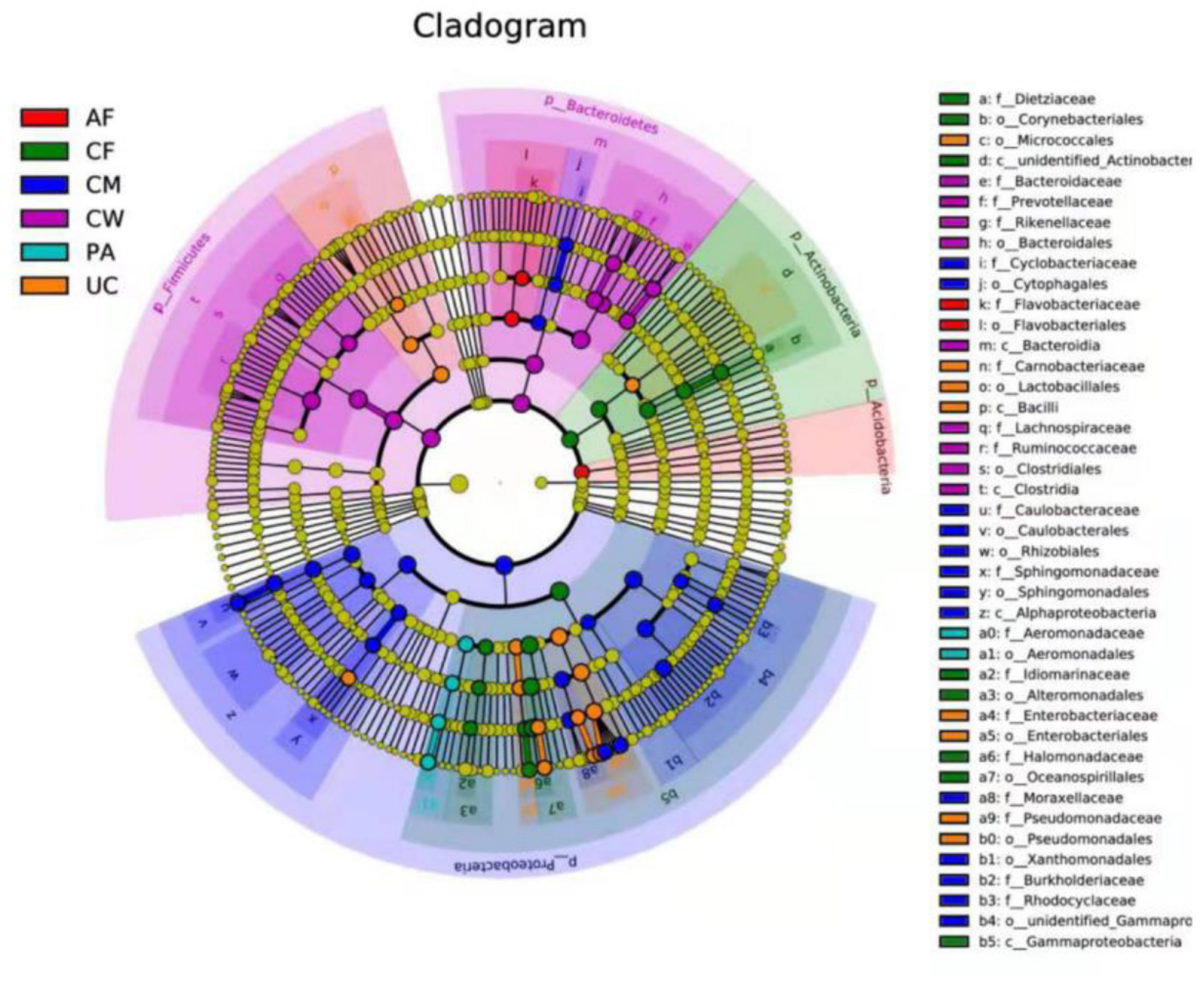
LEfSe-derived phylogenetic trees of the microbiome in all different sample types. AF, amniotic fluid; CF, calf meconium; CM, colostrum; CW, cow feces; PA, placenta; UC, umbilical cord.

LEfSe analysis identified biomarkers with statistically significant differences among different sample types, which can be represented by the LDA score. As shown in Figure 5, 42 microorganisms at different taxonomic levels had LDA scores > 4. The microflora constituents with significant differences in abundance (largest LDA score) between the meconium and other sample types were *Gammaproteobacteria, Oceanospirillales, Halomonadaceae, Halomonas, Actinobacteria, unidentified_Actinobacteria, Corynebacteriales, Dietziaceae, Dietzia, unidentified_Enterobacteriaceae, Alteromonadales, Idiomarinaceae*, and *Aliidiomarina*, and *Gammaproteobacteria*.

### Source Tracing Analysis of the Microbiome Community in the Meconium and Different Maternal Sample Types

#### Overall Source Tracing Analysis of the Microbiome Community in the Meconium and Different Maternal Samples

**Figure 6:**
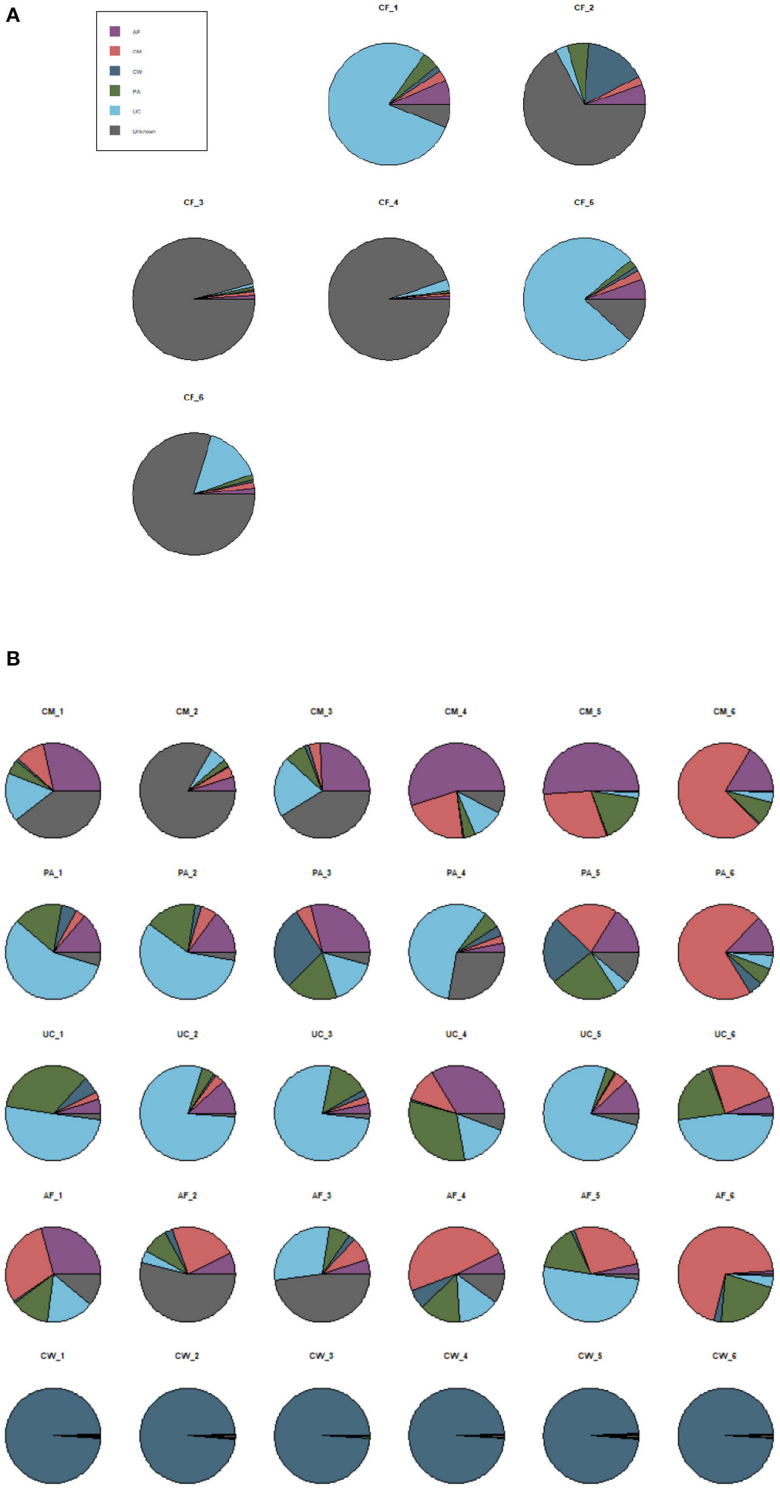
SourceTracker proportion estimates for a subset of “sink” samples and different “source” samples. **(A)** The matching ratio of microorganisms between meconium and different maternal sample types. **(B)** The matching ratio among the five “source” environments. AF, amniotic fluid; CF, calf meconium; CM, colostrum; CW, cow feces; PA, placenta; UC, umbilical cord; Unknown, unknown parts.

SourceTracker software predicted the source of microbial communities in the input sample set, with meconium as the “sink” and the other sample types as different “sources.” The matching ratio of the meconium to other sample types was ordered from high to low for the umbilical cord (23.8 ± 2.21%), placenta (15.57 ± 2.2%), colostrum (14.4 ± 1.9%), amniotic fluid (11.2 ± 1.7%), and cow feces (10.5 ± 1%) (Figure 6A).

SourceTracker software was also used to compare the microorganisms in the five sample types as “sources.” Figure 6B shows that, compared with the other sample types (the autologous microorganism structural characteristics were similar to those in other sample types), the specificities were not obvious in the colostrum, placenta, umbilical cord, and amniotic fluid. However, the specificity between the cow feces and the other sample types was significant.

#### Source Tracing Analysis of the Microbial Community in the Meconium and Different Maternal Sample Types at the Phylum and Genus Levels

SourceTracker software was used to carry out traceability analysis for each cow-calf pair at the phylum and genus levels according to the different floras. This allowed further exploration of the matching ratio of dominant microbiome constituents between the meconium and different maternal sample types. The flora constituents that appeared in the meconium samples of the six calves were selected to ensure the significance of traceability analysis.

**Figure 7:**
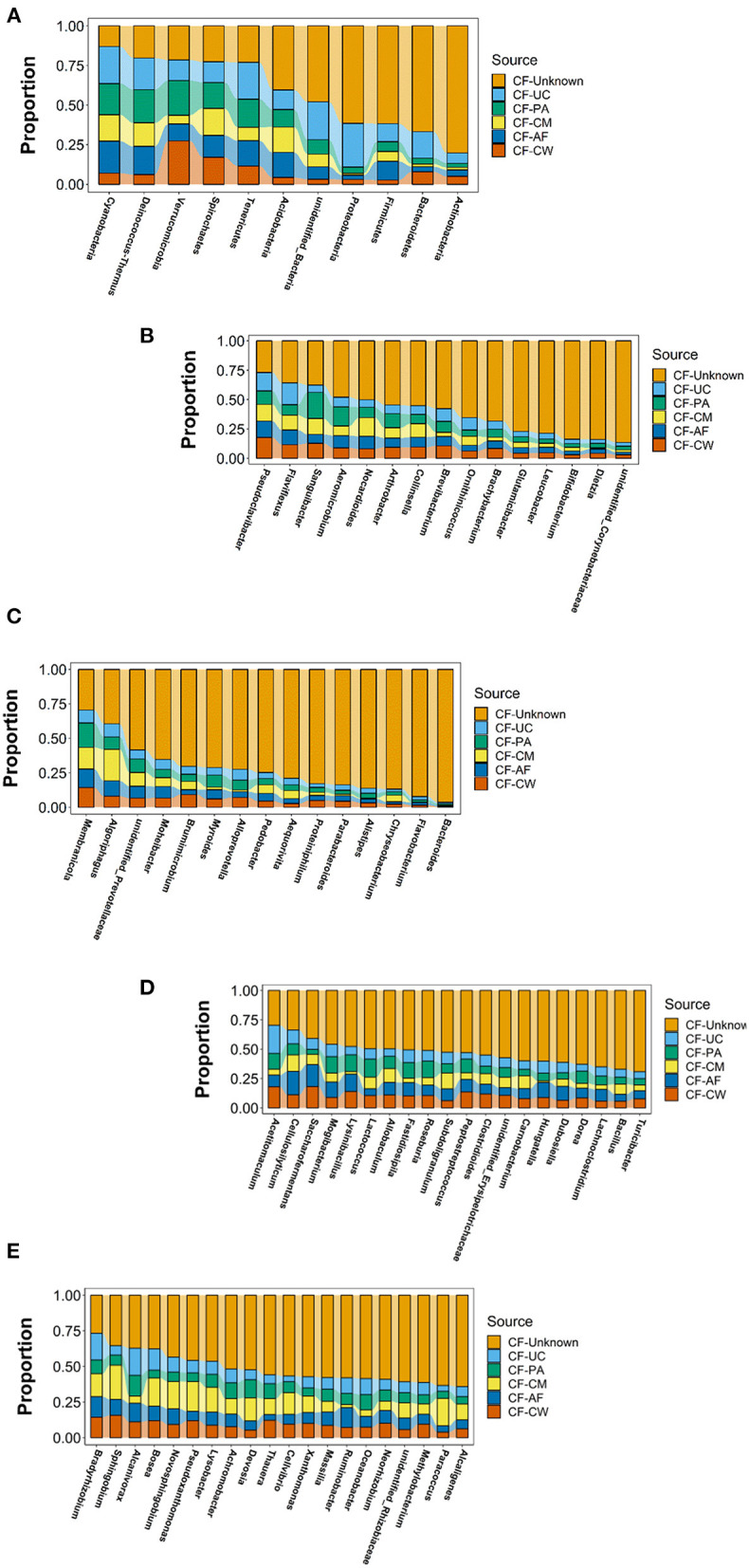
Estimation of the matching ratio of meconium constituents to the main genera in each maternal sample type during maternal transmission. **(A)** Estimation of the matching proportion of the major microbiome constituents at the phylum level. **(B)** Estimation of the matching proportion of major microbiome constituents in Actinobacteria. **(C)** Estimation of the matching proportion of major microbiome constituents in Bacteroidetes. **(D)** Estimation of the matching proportion of major microbiome constituents in Firmicutes (selection of matching proportion in the top 20). **(E)** Estimation of the matching proportion of major microbiome constituents in Proteobacteria (selection of matching proportion in the top 20). AF, amniotic fluid; CF, calf meconium; CM, colostrum; CW, cow feces; PA, placenta; UC, umbilical cord; Unknown, unknown parts.

After screening, a total of 11 phyla, *Cyanobacteria, Deinococcus-Thermus, Verrucomicrobia, Spirochaetes, Tenericutes, Acidobacteria, Proteobacteria, Firmicutes, Bacteroidetes, Actinobacteria*, and *unidentified_Bacteria*, were observed in the maternal transmission process at the phylum level. At the genus level, there were 118 types of major genera in each maternal sample type during the maternal transmission process, which were distributed in *Actinobacteria* (15 genera), *Bacteroidetes* (15 genera), *Firmicutes* (39 genera), *Proteobacteria* (44 genera), *Cyanobacteria* (1 genus), *Deinococcus-Thermu*s (1 genus), *Verrucomicrobia* (1 genus), and *unidentified_Bacteria* (2 genera). Figure 7 presents the main phyla and genera observed during maternal transmission, and the detailed data can be found in Table 5.

**Table 5:** Estimation of the matching ratio of meconium to the main genera in each part of their mothers during maternal transmission.

**Phylum**	**Genus**	**CF-UC**	**CF-PA**	**CF-CM**	**CF-AF**	**CF-CW**	**CF-Unknown**
*Actinobacteria*	*Pseudoclavibacter*	0.1576	0.1153	0.1410	0.1396	0.1785	0.2681
	*Flaviflexus*	0.1853	0.0889	0.1272	0.1239	0.1166	0.3582
	*Sanguibacter*	0.0628	0.2233	0.1343	0.0759	0.1279	0.3758
	*Aeromicrobium*	0.0828	0.1627	0.0819	0.1041	0.0892	0.4793
	*Nocardioides*	0.0647	0.0872	0.1585	0.1077	0.0808	0.5011
	*Arthrobacter*	0.0742	0.1197	0.0864	0.0786	0.0951	0.5460
	*Collinsella*	0.0731	0.0800	0.1144	0.0849	0.0954	0.5522
	*Brevibacterium*	0.1080	0.0936	0.0355	0.0788	0.1071	0.5769
	*Ornithinicoccus*	0.1043	0.0536	0.0772	0.0495	0.0612	0.6542
	*Brachybacterium*	0.0673	0.0697	0.0319	0.0634	0.0844	0.6833
	*Glutamicibacter*	0.0458	0.0462	0.0462	0.0474	0.0439	0.7705
	*Leucobacter*	0.0485	0.0363	0.0345	0.0466	0.0473	0.7867
	*Bifidobacterium*	0.0376	0.0321	0.0320	0.0302	0.0314	0.8367
	*Dietzia*	0.0339	0.0384	0.0098	0.0368	0.0428	0.8384
	*unidentified_Corynebacteriaceae*	0.0334	0.0320	0.0195	0.0213	0.0305	0.8633
*Bcteroidetes*	*Membranicola*	0.0944	0.1764	0.1569	0.1347	0.1431	0.2944
	*Algoriphagus*	0.0957	0.0899	0.2292	0.1112	0.0801	0.3939
	*unidentified_Prevotellaceae*	0.0669	0.0973	0.1003	0.0858	0.0661	0.5837
	*Moheibacter*	0.0713	0.0614	0.0644	0.0829	0.0673	0.6527
	*Brumimicrobium*	0.0578	0.0520	0.0599	0.0346	0.0927	0.7029
	*Myroides*	0.0559	0.0861	0.0184	0.0676	0.0605	0.7115
	*Alloprevotella*	0.0790	0.0712	0.0125	0.0420	0.0715	0.7237
	*Pedobacter*	0.0432	0.0467	0.0619	0.0563	0.0447	0.7472
	*Aequorivita*	0.0469	0.0417	0.0588	0.0355	0.0269	0.7903
	*Proteiniphilum*	0.0276	0.0354	0.0234	0.0362	0.0489	0.8284
	*Parabacteroides*	0.0385	0.0289	0.0125	0.0381	0.0445	0.8375
	*Alistipes*	0.0359	0.0351	0.0053	0.0289	0.0324	0.8624
	*Chryseobacterium*	0.0191	0.0239	0.0483	0.0176	0.0225	0.8686
	*Flavobacterium*	0.0244	0.0180	0.0014	0.0190	0.0151	0.9220
	*Bacteroides*	0.0102	0.0098	0.0009	0.0080	0.0068	0.9644
*Firmicutes*	*Acetitomaculum*	0.2396	0.1344	0.0500	0.1002	0.1793	0.2965
	*Cellulosilyticum*	0.1175	0.0950	0.1400	0.1992	0.1117	0.3367
	*Saccharofermentans*	0.0937	0.0421	0.0876	0.1869	0.1815	0.4081
	*Mogibacterium*	0.1053	0.1402	0.0759	0.1308	0.0895	0.4583
	*Lysinibacillus*	0.0713	0.1435	0.0213	0.1472	0.1398	0.4769
	*Lactococcus*	0.0891	0.1525	0.0989	0.0580	0.1056	0.4960
	*Allobaculum*	0.0643	0.1041	0.1176	0.1073	0.1101	0.4965
	*Fastidiosipila*	0.1086	0.1324	0.0375	0.1129	0.1027	0.5058
	*Roseburia*	0.0915	0.1399	0.0639	0.0885	0.1057	0.5105
	*Subdoligranulum*	0.0975	0.0793	0.1340	0.1014	0.0615	0.5263
	*Peptostreptococcus*	0.0556	0.1166	0.0565	0.1069	0.1355	0.5290
	*Clostridioides*	0.0943	0.0636	0.0899	0.0829	0.1191	0.5502
	*unidentified_Erysipelotrichaceae*	0.0831	0.0817	0.0886	0.0658	0.1069	0.5737
	*Carnobacterium*	0.0718	0.0553	0.1086	0.0858	0.0789	0.5996
	*Hungatella*	0.1011	0.0664	0.0126	0.1284	0.0889	0.6026
	*Dubosiella*	0.0877	0.0538	0.0619	0.1192	0.0648	0.6126
	*Dorea*	0.0590	0.1039	0.0406	0.0828	0.0854	0.6283
	*Lachnoclostridium*	0.0786	0.0747	0.0385	0.1004	0.0576	0.6502
	*Bacillus*	0.0668	0.0596	0.0861	0.0598	0.0569	0.6708
	*Turicibacter*	0.0587	0.0542	0.0485	0.0689	0.0775	0.6922
*Proteobacteria*	*Sphingobium*	0.0654	0.0712	0.2400	0.1118	0.1572	0.3545
	*Bradyrhizobium*	0.1872	0.0966	0.1607	0.1444	0.1441	0.2670
	*Thauera*	0.0629	0.1047	0.1111	0.0406	0.1224	0.5584
	*Pseudoxanthomonas*	0.0879	0.0613	0.2083	0.0655	0.1197	0.4572
	*Bosea*	0.1500	0.0544	0.1976	0.1019	0.1197	0.3764
	*Alcanivorax*	0.1903	0.1455	0.0500	0.1306	0.1115	0.3721
	*Neorhizobium*	0.0752	0.0786	0.0650	0.0895	0.1016	0.5902
	*Xanthomonas*	0.0789	0.0570	0.1149	0.0764	0.1007	0.5722
	*Cellvibrio*	0.0399	0.0774	0.1508	0.0693	0.0954	0.5671
	*Methylobacterium*	0.0866	0.0639	0.0721	0.0699	0.0953	0.6122
	*Novosphingobium*	0.1056	0.0658	0.1909	0.1099	0.0934	0.4343
	*Lysobacter*	0.0922	0.0905	0.1736	0.0927	0.0877	0.4634
	*Massilia*	0.0810	0.0855	0.0745	0.0944	0.0863	0.5783
	*Achromobacter*	0.0970	0.1112	0.1079	0.0891	0.0770	0.5177
	*Oceanobacter*	0.1126	0.1070	0.0447	0.0763	0.0743	0.5852
	*Ruminobacter*	0.1089	0.0789	0.0205	0.1394	0.0722	0.5800
	*Alcaligenes*	0.0703	0.0517	0.1111	0.0627	0.0627	0.6414
	*unidentified_Rhizobiaceae*	0.0763	0.0711	0.1061	0.0811	0.0575	0.6080
	*Devosia*	0.0694	0.1273	0.1612	0.0649	0.0537	0.5236
	*Paracoccus*	0.0411	0.0492	0.1915	0.0450	0.0394	0.6337
*Cyanobacteria*	*unidentified_Cyanobacteria*	0.0748	0.0701	0.0640	0.0767	0.0535	0.6609
*Deinococcus-Thermus*	*Truepera*	0.0440	0.0597	0.2087	0.0698	0.0826	0.5352
*unidentified_Bacteria*	*Helicobacter*	0.1221	0.1183	0.1295	0.0947	0.1086	0.4268
	*Arcobacter*	0.0785	0.0908	0.0005	0.0603	0.0573	0.7125
*Verrucomicrobia*	*Akkermansia*	0.0605	0.0828	0.0134	0.0596	0.0732	0.7106

*CF-UC, matching ratio of meconium to umbilical cord; CF-PA, matching ratio of meconium to placenta; CF-CM, matching ratio of meconium to colostrum; CF-AF, matching ratio of meconium to amniotic fluid; CF-CW, matching ratio of meconium to cow feces; CF-Unknown, matching ratio of meconium to unknown parts*.

## Discussion

Several studies have previously investigated the presence of microorganisms in the prenatal fetal gut and compared the offspring hindgut microbiome with that from different maternal sites such as the vagina, colostrum, and maternal feces (5, 6, 18, 30). Studies used healthy mare-foal, ewe-lamb, and cow-calf pairs as animal models and concluded that the prenatal gut harbored active microorganisms and that the fetal gut microbiome was seeded antenatally (7, 31). Regarding the hindgut microflora of calves, the current literature indicates that microbial communities across hindgut segments differ (32, 33). Thus, by examining the microbiome in meconium and different maternal sample types we were able to explore the possible sources of the hindgut microbiome in newborn calves. To obtain repeat and subsequent samples without euthanasia, a non-invasive and practical method (i.e., meconium examination) was chosen. The meconium microbiome represented the gut microbiome at birth without environmental influences such as feeding.

Meconium is the feces present in the hindgut of the calf before birth and can largely reflect the condition of the fetus' intestinal flora in the mother's womb. Both, the placenta after birth. Amniotic fluid is the only environment for the fetus to survive in the mother's womb and the placenta and umbilical cord are important ways for the mother to transfer nutrients to the fetus and for the fetus to metabolize them. This suggests that the similarity of the early fetal gut flora structure to its environment at the phylum level can reveal important information about the origin of microbial colonization *in utero*.

The bacterial microbiome of the samples from each site was analyzed using the Illumina Nova sequencing platform. At the phylum level, the dominant microbial components of the placenta, umbilical cord, amniotic fluid, colostrum, meconium, and cow feces included *Proteobacteria, Firmicutes, Bacteroidetes*, and *Actinobacteria*.

*Proteobacteria* are gram-negative bacteria that can produce LPS (lipopolysaccharide) that can enter the blood, reduce the number of hindgut barrier cells, and increase hindgut permeability (34). *Bacteroidetes* produce butyrate, a product of colonic fermentation with antineoplastic properties, and are beneficial to interactions within the immune system of the host, which can activate T cell-mediated responses (35) and limit the colonization of potentially pathogenic bacteria in the GI tract (35). A previous study found that Proteobacteria, which colonized the intestines of young mice at an early stage, can activate the young mouse's immune system. The high levels of Proteobacteria in meconium in this study may also be related to the construction of the early immune system in calves (36). Thus, the presence of these bacteria in the hindgut of the neonatal calf is advantageous in the context of health (37). *Actinobacteria* can use carbohydrates to produce lactic acid, which can maintain the acidity of the environment and suppress the growth of pathogenic bacteria in the intestine (38). In addition, microbiota-depleted mice inoculated with Bacteroidetes and Firmicutes reflected that the two phyla both have distinct effects on hindgut immunity by differentially inducing primary and secondary response genes (39).

Similar to previous studies (18, 19, 30), the results in the present study indicated that the core community in meconium was enriched in *Halomonadaceae, Pseudomonadaceae, Dietziaceae, Ruminococcaceae, Moraxellaceae*, and *Enterobacteriaceae*. These responses were also similar to those in human studies (40). Accordingly, we speculate that the microbiome in the abovementioned core community is the “pioneer flora” during early life of the offspring. As such, it plays a central role in shaping the community of anaerobic organisms. Results from other studies (19, 30) can also be corroborated by differences in the composition of the microbiome between meconium and cow feces in the present study: compared with those in newborn calves, *Proteobacteria* levels were reduced in young and adult calves, and *Firmicutes* and *Bacteroides* dominated the fecal microbiome (33). These results indicated that the increasing diversity and richness in hindgut microbiome communities as the animal ages are indicative of progressive establishment of a complex microbiome during early life stages (33).

The presence of *Pseudomonas, Limnobacter*, and *Brevundimonas* as the most abundant genera in colostrum underscored the key role of colostrum in helping colonize the neonatal gut with microflora with obvious beneficial effects (e.g., probiotic effect) (41). *Pseudomonas* has been consistently reported to be the dominant microbe in colostrum (42) and raw milk (43). Li et al. (44) concluded that *Pseudomonas, Lactococcus*, and *Acinetobacter* were the most common genera, and Hang et al. (45) found that *Streptococcus, Acinetobacter, Enterobacter*, and *Corynebacterium* were the dominant microbiota. Although those studies differed from our results, it is possible that differences in experimental approaches and even environment account for most of the discrepancies. For example, Hang et al. (45) squeezed colostrum samples into a non-sterile bucket and collected them directly from the bucket after mixing. Clearly, use of a non-sterile container likely would have contaminated the samples.

Transmission of microbiota from milk to the developing offspring may exert many short- or long- term influences on the physiology of the offspring (46). For example, Lactobacilli in milk include species associated with the hindgut microbiome (47). These microorganisms can produce a large quantity of lactic acid, which can inhibit the growth of pathogenic bacteria (38). Lactic acid can also be converted to butyrate, which maintains the acidity of the environment and suppresses growth of pathogens in the intestine (48). In addition, it has been documented that flora in the maternal gut can reach the mammary gland *via* intestinal mononuclear cells during late gestation and lactation, also suggesting the existence of bacterial transmission *via* intestinal-lacteal routes (46). The offspring's intestinal microbiota and its immune evolution are related to milk microbiota, which are derived from the maternal entero-mammary pathway (49). Interestingly, although calves did not have access to the udder at the time of sampling in this experiment, at the phylum level the colostrum flora matched the meconium flora by 14.4%, which was very close to the match between the placental flora and the meconium flora (15.5%). This suggested that colostrum microorganisms have some influence on meconium. DiGiulio et al. (50) suggested that the perinatal transfer of beneficial microorganisms from the maternal gut to the mammary gland *via* the bloodstream, i.e., the adjustment of the oligo-oligosaccharides, immune factors and microbial communities in milk before delivery helped prepared, so that these prepared “beneficial bacteria” for transmission to the offspring through milk after birth. This may be a specific evolutionary phenomenon.

Quercia et al. (6) concluded that amniotic fluid and intestinal ecosystems can also contribute uniquely to the meconium microbiome community in foals. He et al. (4) studied the association of the microbiome in infant meconium with that in maternal vagina, saliva, amniotic fluid, and feces samples. Their data indicated that the meconium microbiome was seeded from multiple maternal body sites, with amniotic fluid microbiome contributing the most. Thus, vertical transmission of the microbiome from the mother to the offspring may exist. A hypothetical “enteromammary” pathway was proposed in which the selected bacteria in the maternal intestine can access the mammary glands, and dendritic cells and CD18+ cells can take up non-pathogenic bacteria from gut epithelial cells and carry them to other locations (51). The placental microbiome in mice is colonized by invasion and crossing of the endothelial lining (52), a process thought to occur during early vascularization and placentation (15). Dendritic cells from the mare penetrate the host epithelia including hindgut epithelium carrying luminal bacteria or bacterial antigens that are then released into the placenta *via* the bloodstream (6). Once the amniotic fluid is reached, these microbial factors may have access to the fetal gut and become a part of the meconium ecosystem (53). Various bacteria can also be released into the breast through the blood. Thus, we speculate that the main biological function of microbial factor transfer from the intrauterine region to the fetus may be beneficial to the development of digestive function and the construction of the immune system of newborn calves after delivery.

SourceTracker analysis showed that in the maternal transmission process, cow feces mainly transmitted acid-producing bacteria such as *Saccharofermentans, Acetitomaculum*, and *Pseudoclavibacter*. *Saccharofermentans* are fibrolytic (54) and produce short-chain fatty acids and low-density lipoprotein cholesterol (6) both of which help maintain health and provide energy for the developing intestinal wall (55). *Acetitomaculum* and *Pseudoclavibacter* produce mainly acetic acid and butyric acid, respectively (55, 56). Butyrate, as an energy source for host epithelial cells, can regulate growth and the differentiation-related activator protein 1 (AP-1) signaling pathway (57) leading to an increase in the number of immunoregulatory T regulatory (T-reg) cells. These functions may reduce the likelihood of maternal rejection of the fetal allograft (58).

Other groups of bacteria that appear mainly transmitted from cow feces are common in the digestive tract and are associated with nutrition such as the aerobic denitrification bacteria *Thauera, Lysinibacillus*, and *Peptostreptococcus* and *Novosphingobium*, which are core members of the gut flora in the cow (18). *Cellulosilyticum, Saccharofermentans*, and *Ruminobacter*, the main cellulose-degrading bacteria (59, 60), and *Bradyrhizobium, Mogibacterium, Alcanivorax, Fastidiosipila, Saccharofermentans*, and *Ruminobacter*, are all common bacteria in alimentary canals involved in fiber degradation (59, 61, 62). These are all key microbiome communities transmitted by amniotic fluid. In fact, these bacteria were the main transmitted bacteria that were not only in cow feces and amniotic fluid, but also in placenta and umbilical cord and dominated in the succession that occurred in early life (58). *Algoriphagus, Pseudoxanthomonas, Bradyrhizobium*, and *Novosphingobium* were the main cellulose-degrading microbiome constituents (63–65) transmitted *via* colostrum. In addition, it was reported that the special genus *Truepera*, which was mainly transmitted by colostrum, was the core flora constituent in bedding used to house cows (66).

Overall, microbial communities of the cow-calf pair encompassed a complex and shared microbiome that likely interacted to maintain health in both cows and calves (40, 67). Although there were differences in microbial community structure among different sample types of dams and offspring, the microbiome involved in cellulose degradation, fermentation, and the common flora in alimentary canals were seeded into the calf *via* the maternal transmission process and affected the calf's nutrition and the microbial communities existing in the calf intestine.

Some limitations in the present study must be mentioned. First, the number of cow-calf pairs could be considered small for a robust evaluation of maternal transmission. Second, a deviation in PCR results may have occurred in the analysis of low-microbial-biomass samples, and the possibility of contamination of samples cannot be completely excluded. Third, the inherent limits of molecular analyses did not allow for studying whether live or dead bacteria, even microbial debris, were present in the samples collected. Lastly, we identified numerous flora in the meconium, but the origins and timing of the colonization were not investigated, and knowledge about the influence of these microflora on the metabolism and immune function remains limited.

## Conclusion

Data provide evidence that the fetal hindgut microbiome of the calf may arise from different maternal parts. The composition of the meconium microflora originated from multiple maternal sites including umbilical cord, placenta, colostrum, and amniotic fluid. Characteristics of the microorganisms in the placenta, umbilical cord, colostrum, and meconium were more obvious than those in amniotic fluid, and differences in the microbial characteristics between meconium and cow feces were the largest. Microflora with digestive functions such as cellulose decomposition and rumen fermentation were highly matched during the maternal transmission process. Overall, the present findings advanced our understanding of the calf gut microbiome and lays a foundation for improving the growth and development of offspring, hindgut health, and lactation potential of calves by intervening in the gut microecology of pregnant cows. Further studies are required to gain an in-depth understanding of the origin, composition, function, dynamics, and colonization time of the calf gut microbiome. Elucidating the effects of the fetal gut microbiome on development, immunity, and health throughout early life will be an important undertaking.

## Data Availability Statement

The raw data supporting the conclusions of this article will be made available by the authors, without undue reservation. The data presented in the study are deposited in the NCBI BioProject dataset repository, and the BioProject ID is PRJNA768139.

## Ethics Statement

The animal study was reviewed and approved by Animal Welfare and Ethics Committee of Heilongjiang Bayi Agriculture University.

## Author Contributions

HZ, CX, and YQ: conceptualization, methodology, and supervision. MY and SY: experiments and data curation. HZ and LL: writing-original draft preparation and software. JL, AE, and WW: reviewing and editing. All authors read and approved the final version of the manuscript.

## Funding

This research was funded by the National Natural Science Foundation of China (Grant Nos. 32072758, U20A2062, and 31902186), the Natural Science Foundation of Heilongjiang Province of China (LH2021C069), and the Scientific Research Starting Foundation for Returned Overseas Chinese Scholars (Grant No. ZRCLG201903).

## Conflict of Interest

The authors declare that the research was conducted in the absence of any commercial or financial relationships that could be construed as a potential conflict of interest.

## Publisher's Note

All claims expressed in this article are solely those of the authors and do not necessarily represent those of their affiliated organizations, or those of the publisher, the editors and the reviewers. Any product that may be evaluated in this article, or claim that may be made by its manufacturer, is not guaranteed or endorsed by the publisher.
