# Supplementary material for: Analysis of Cow-Calf Microbiome Transfer Routes and Microbiome Diversity in the Newborn Holstein Dairy Calf Hindgut
Source: Front Nutr. 2021 Oct 25;8:736270. doi: 10.3389/fnut.2021.736270 (PMC8573054; doi:10.3389/fnut.2021.736270)
Supplement: Supplementary file 1 [file Table_1.docx]

**Table S1 Sample information, microbial diversity, and sequence abundance at genus level.**

| **Group** | **Sample** | **Effective Tags** | **Number of Reads** | **Observed_species** | **Shannon** | **Simpson** | **Chao1** | **ACE** |
| --- | --- | --- | --- | --- | --- | --- | --- | --- |
| PA | PA1 | 65,383 | 107,121 | 3033 | 9.225 | 0.994 | 3413.149 | 3596.613 |
|  | PA2 | 63,761 | 100,806 | 2622 | 8.928 | 0.989 | 2930.521 | 2994.948 |
|  | PA3 | 63,094 | 103,571 | 2706 | 9.076 | 0.994 | 3042.125 | 3206.087 |
|  | PA4 | 60,211 | 102,137 | 2286 | 7.177 | 0.941 | 2617.139 | 2750.013 |
|  | PA5 | 56,283 | 80,619 | 3089 | 8.899 | 0.988 | 3552.014 | 3628.601 |
|  | PA6 | 60,968 | 90,016 | 1467 | 5.741 | 0.901 | 1674.24 | 1792.744 |
| UC | UC1 | 65,164 | 103,997 | 2457 | 8.61 | 0.99 | 2824.511 | 3000.616 |
|  | UC2 | 66,350 | 111,306 | 2511 | 6.935 | 0.918 | 3372.085 | 3359.719 |
|  | UC3 | 67,531 | 107,816 | 2364 | 7.737 | 0.975 | 3043.76 | 2982.242 |
|  | UC4 | 64,881 | 108,798 | 2277 | 7.487 | 0.964 | 2554.028 | 2714.346 |
|  | UC5 | 46,685 | 79,170 | 2730 | 7.502 | 0.972 | 3672.453 | 3852.258 |
|  | UC6 | 62,463 | 84,655 | 1492 | 6.658 | 0.962 | 1682.691 | 1748.391 |
| AF | AF1 | 67,504 | 107,284 | 4463 | 10.002 | 0.996 | 5088.179 | 5434.284 |
|  | AF2 | 65,884 | 110,136 | 4082 | 9.595 | 0.993 | 4654.656 | 4831.078 |
|  | AF3 | 47,284 | 72,785 | 3214 | 8.549 | 0.978 | 3682.214 | 3759.361 |
|  | AF4 | 68,112 | 112,560 | 3349 | 7.934 | 0.95 | 3778.5 | 4017.159 |
|  | AF5 | 48,619 | 75,203 | 1815 | 7.501 | 0.977 | 2085.473 | 2162.079 |
|  | AF6 | 67,006 | 98,994 | 1146 | 4.569 | 0.847 | 1547.049 | 1570.494 |
| CM | CM1 | 67,278 | 105,273 | 3590 | 7.393 | 0.931 | 4195.968 | 4512.558 |
|  | CM2 | 62,369 | 98,128 | 2569 | 8.352 | 0.985 | 2981.427 | 3211.042 |
|  | CM3 | 66,482 | 102,904 | 3359 | 9.022 | 0.992 | 3943.359 | 4132.406 |
|  | CM4 | 60,657 | 97,567 | 3959 | 9.348 | 0.987 | 4622.621 | 4785.536 |
|  | CM5 | 60,601 | 87,110 | 1098 | 3.656 | 0.777 | 1318.487 | 1419.595 |
|  | CM6 | 62,493 | 88,187 | 843 | 3.837 | 0.819 | 979.906 | 1060.933 |
| CW | CW1 | 61,661 | 80,071 | 1139 | 7.646 | 0.986 | 1237.456 | 1255.693 |
|  | CW2 | 60,847 | 86,098 | 1315 | 8.026 | 0.986 | 1405.144 | 1434.665 |
|  | CW3 | 62,501 | 82,375 | 1256 | 7.844 | 0.985 | 1337.54 | 1373.56 |
|  | CW4 | 63,492 | 98,246 | 1318 | 7.896 | 0.985 | 1438.347 | 1471.439 |
|  | CW5 | 66,663 | 93,401 | 1277 | 7.839 | 0.985 | 1485.496 | 1447.223 |
|  | CW6 | 61,438 | 68,425 | 1147 | 7.279 | 0.98 | 1256.073 | 1282.679 |
| CF | CF1 | 69,338 | 113,490 | 1570 | 7.46 | 0.977 | 1967.872 | 1919.5 |
|  | CF2 | 63,218 | 96,077 | 2308 | 8.191 | 0.985 | 2583.321 | 2693.786 |
|  | CF3 | 67,655 | 108,127 | 877 | 3.004 | 0.627 | 1183.843 | 1113.445 |
|  | CF4 | 69,701 | 108,552 | 535 | 2.832 | 0.678 | 843.812 | 895.146 |
|  | CF5 | 62,320 | 68,064 | 1519 | 6.783 | 0.956 | 1706.289 | 1769.628 |
|  | CF6 | 64,552 | 108,141 | 1078 | 3.782 | 0.625 | 1272.752 | 1297.366 |

PA: placenta; UC: umbilical cord; AF: amniotic fluid; CM: colostrum; CW: cow feces; CF: calf meconium; Effective Tags: the final Tags sequence for subsequent analysis after filtering chimera; Number of reads: PE reads of original offline
