# Supplementary material for: Analysis of Cow-Calf Microbiome Transfer Routes and Microbiome Diversity in the Newborn Holstein Dairy Calf Hindgut
Source: Front Nutr. 2021 Oct 25;8:736270. doi: 10.3389/fnut.2021.736270 (PMC8573054; doi:10.3389/fnut.2021.736270)
Supplement: Supplementary file 3 [file Table_3.docx]

**Table S3 Operational taxonomic units (OTUs) found exclusively in one type of sample at genus level.**

| **Placenta** | **Umbilical bord** | **Amniotic fluid** | **Colostrum** | **Calf meconium** | **Cow feces** |
| --- | --- | --- | --- | --- | --- |
| *Candidatus_Tenderia* | *Methyloglobulus* | *Halochromatium* | *Tunicatimonas* | *Halorubrum* | *Syntrophorhabdus* |
| *Paramaledivibacter* | *Paraperlucidibaca* | *unidentified_Nitrospirae* | *Pelagibius* | *Desulfitobacterium* | *Defluviitoga* |
| *Marinifilum* | *Thermanaerothrix* | *Merdibacter* | *Fervidobacterium* | *Senegalimassilia* | *Pelotomaculum* |
| *Aeribacillus* | *Thalassotalea* | *Desulfobacula* | *unidentified_*  *Leptotrichiaceae* | *Wenzhouxiangella* | *Candidatus_*  *Cloacimonas* |
| *Euzebya* | *Lacihabitans* | *unidentified_Candidatus_*  *Peregrinibacteria* | *unidentified_*  *Rhodospirillaceae* | *Egicoccus* |  |
| *Larkinella* | *Myceligenerans* | *Draconibacterium* | *Candidatus_*  *Odyssella* | *Gallibacterium* |  |
| *unidentified_*  *Cryomorphaceae* | *Robiginitomaculum* | *Catabacter* | *Entomoplasma* | *Chlamydia* |  |
| *Kordiimonas* | *Candidatus_Kuenenia* | *Endobacter* | *Rhizocola* | *unidentified_Anaerolineaceae* |  |
| *Schleiferia* | *unidentified_*  *Marinilabiliaceae* | *Aquaspirillum* | *Candidatus_*  *Schmidhempelia* | *Leptonema* |  |
| *Stackebrandtia* | *Motilimonas* | *Williamsia* | *Mariniphaga* | *Lewinella* |  |
| *Prosthecochloris* | *unidentified_*  *Ktedonobacteraceae* | *Fontibacter* | *Sporacetigenium* | *Alicyclobacillus* |  |
| *Salinispirillum* | *Olivibacter* | *Wandonia* | *Synergistes* | *Permianibacter* |  |
| *Geminicoccus* | *Thalassospira* | *Methylocaldum* | *Symbiobacterium* | *Thermicanus* |  |
| *Raoultibacter* | *Aeriscardovia* | *Glaciecola* | *Halofilum* | *Thermodesulfobacterium* |  |
| *Oligosphaera* | *Enorma* | *Thioalkalispira* | *Leptospirillum* | *Sulfurospirillum* |  |
| *Aquimarina* | *Ethanoligenens* | *unidentified_Phycisphaerae* | *Psychromonas* | *Verrucomicrobium* |  |
| *Leucothrix* | *Thermobaculum* | *unidentified_Cytophagales* | *Thermobrachium* | *unidentified_*  *Methylacidiphilaceae* |  |
| *unidentified_*  *Cellvibrionales* | *Salegentibacter* | *Candidatus_Finniella* | *Candidatus_*  *Latescibacter* | *Leadbetterella* |  |
| *Alkanibacter* | *Taeseokella* | *Desulforhabdus* | *Candidatus_*  *Protochlamydia* | *Quinella* |  |
| *Proteiniborus* | *Rickettsia* | *Oceaniserpentilla* | *Oceanococcus* | *Fimbriiglobus* |  |
| *Iodidimonas* | *Desulfotomaculum* | *Pullulanibacillus* | *Hahella* |  |  |
| *Anaerofilum* |  | *Oscillochloris* | *unidentified_*  *Sphingomonadaceae* |  |  |
| *Desulfocarbo* |  |  |  |  |  |
| *Cenarchaeum* |  |  |  |  |  |
| *unidentified_*  *Actinomarinales* |  |  |  |  |  |
| *Breznakia* |  |  |  |  |  |
